# Safety and Efficacy of Hypertonic Sodium Chloride 5% Ointment for Recurrent Corneal Erosion Syndrome

**DOI:** 10.7759/cureus.32796

**Published:** 2022-12-21

**Authors:** Michael Tsatsos, Artemis Matsou, Marinos Soultanidis, Ioannis K Athanasiadis

**Affiliations:** 1 Specialist Ophthalmology Services, Ophthalmore, Thessaloniki, GRC; 2 Ophthalmology, Queen Victoria Hospital, London, GBR; 3 Ophthalmology, Moorfields Eye Centre at Bedford, Bedford, GBR

**Keywords:** visual acuity, endothelium, cornea, recurrent erosion, 5% sodium chloride ointment

## Abstract

Background

In this study, we aimed to assess the safety and efficacy of hypertonic sodium chloride 5% ointment in the treatment of recurrent corneal erosion syndrome (RCES).

Methodology

A total of 21 eyes from 21 patients with RCES either following trauma or spontaneously due to underlying anterior basement membrane dystrophy (ABMD) were prospectively enrolled over a six-month period. The acute episode was managed with topical sodium chloride 5% ophthalmic ointment applied twice daily for 30 days. Patients were followed up at one month and six months with visual acuity and endothelial cell count (ECC) measurement.

Results

The mean age was 44.19 years (range = 17-87 years). All patients had unilateral involvement. The etiology was ABMD in 12 cases, while nine cases were post-traumatic. The mean best-corrected visual acuity (BCVA) at presentation was 0.32 logMAR units (SD = 0.18), and the mean ECC before treatment initiation was 2,720 cells/mm^2 ^(SD ± 192). At the one-month follow-up, all patients had a full recovery with complete re-epithelization of the defective area and resolution of symptoms. The mean BCVA was 0.05 logMAR units (SD = 0.12), and the mean ECC was 2,703 cells/mm^2^ (SD = 205). At six months, only one recurrence was documented following another episode of trauma. The mean BCVA and ECC at six months were 0.01 logMAR units and 2,714 cells/mm^2^, respectively.

Conclusions

Sodium chloride 5% ophthalmic ointment applied twice daily for 30 days following the acute event seems to be a safe and effective treatment option for RCES from both traumatic and ABMD etiology.

## Introduction

Recurrent corneal erosion syndrome (RCES), first reported by Hansen in 1872 [1], is identified as one of the most frequent causes of repeated episodes of sudden-onset ocular pain, occurring usually upon awakening or at night, and remains a relatively common reason for attending an emergency eye clinic. The often disabling symptoms of ocular discomfort, photophobia, blurred vision, redness, and lacrimation are related to the spontaneous de-epithelialization of the cornea due to defective adhesion of the epithelium to the underlying basement membrane [2]. Abnormal hemi-desmosomes or anchoring filaments at the basal layer of the epithelium [2], upregulation of matrix metalloproteinases (MMPs) [3], and an overly tight adherence of the lids to the corneal epithelium [4] have been implicated in the pathophysiology of RCES. Erosions can develop following a superficial corneal injury (post-traumatic), in the context of anterior basement membrane dystrophy (ABMD/Cogan dystrophy or map-dot-fingerprint dystrophy), and in certain systemic disorders such as epidermolysis bullosa and diabetes [5].

Treatment options vary from medical management with topical lubrication, topical antibiotics, cycloplegia, eye patching, bandage contact lenses, oral tetracycline (doxycycline), hypertonic saline ointments [6,7] to surgical treatment modalities such as anterior stromal puncture, alcohol delamination, excimer laser therapy, phototherapeutic keratectomy (PTK), diamond burr polishing, and neodymium-doped yttrium aluminum garnet (Nd-YAG laser) [5,8,9]. Sodium chloride (NaCl) 5% ointment is a hypertonic salt solution used to reduce corneal edema by drawing water out of the cornea, which, in turn, evaporates through the tear film. Currently, it is the second-line option after lubrication in order to treat and prevent recurrences of RCES [9]. It is thought that the sudden reduction in corneal edema could be damaging to the corneal endothelium. With a view to assessing the safety and efficacy of NaCl 5% ointment use in the treatment and prevention of RCES reappearance, we conducted a prospective assessment of our practice.

## Materials and methods

Patients presenting in the Eye Casualty Unit of a tertiary ophthalmic center with recurrent corneal erosion syndrome were prospectively enrolled in our descriptive study over a period of six months. All patients provided written informed consent before enrolment. Institutional ethics committee approval by the Hellenic College of Ophthalmology was obtained (approval number: 03/H0512/14), and the study adhered to the tenets of the Declaration of Helsinki. The inclusion criterion was any patient with RCES from any cause presenting with an acute episode of recurrent corneal erosion, previously managed with any form of conservative treatment including topical lubricants. Patients with prior surgical intervention such as alcohol delamination or PTK for RCES were excluded from the study. A total of 21 eyes from 21 patients were reviewed.

Upon initial presentation, details of sex, age, prior history of ocular trauma and surgery, as well as any ophthalmic and medical diseases were recorded. A careful history of the presenting complaint (onset, duration, nature of symptoms) was obtained from all patients, combined with a comprehensive ophthalmic examination including best-corrected visual acuity (BCVA) and slit lamp biomicroscopy. The diagnosis of corneal erosion was made based on the spontaneous occurrence of a frank focal epithelial defect or an area of a weakly adhered corneal epithelium as seen on slit lamp examination using broad-beam illumination, retroillumination, and cobalt-blue light after fluorescein staining. Endothelial cell counts were obtained in the acute phase for all patients.

The acute episode was managed with topical preservative-free sodium chloride 5% ointment applied twice daily (in the morning and before bedtime) for 30 days. Patients were followed up with slit lamp examination at one month and six months after the acute event. Patients were instructed to return to the corneal acute clinic earlier if they experienced any problems. A complete ophthalmic examination of the anterior segment was performed at the time point and patients were questioned about symptoms of recurrence. Endothelial cell counts were measured at both subsequent follow-up visits (Tomey EM-3000, Tomey Co., Japan).

Two mixed-model analysis of variance (ANOVA) tests were used. LogMAR and cell counts were the within-subject factors each time, while age, gender, and diagnosis were between-subject factors. The power of the study was calculated as 0.8.

## Results

Basic demographic characteristics, BCVA measurements, and endothelial cell counts at baseline, first-months, and six-month visits are summarized in Table 1. The mean age was 44.19 years (SD = 20.64, range = 17-87 years). Females accounted for two-thirds of patients (14 females, seven males). All 21 patients had unilateral involvement. The etiology of recurrent erosions was ABMD in 12 patients with no history of trauma, while nine cases were post-traumatic. Patients in the ABMD group were much younger compared to the post-traumatic group (mean age 39.5 in the ABMD group compared to 50.4 years in the post-traumatic group). All patients were followed up for six months and no dropouts were observed. BCVA was measured at all patient visits on Snellen charts and converted to logMAR units. The mean BCVA at presentation was 0.32 (SD = 0.19) logMAR units. Endothelial cell count was measured at presentation for all patients before initiation of treatment with a mean of 2,720 cells/mm^2^ (SD = 193 cells/mm^2^).

**Table 1:** Summary of baseline demographics, BCVA, and endothelial cell counts at presentation and first-month and six-month follow-ups. M = male; F = female; BCVA = best-corrected visual acuity; ABMD = anterior basement membrane dystrophy

Gender	Diagnosis	BCVA logMar - Presentation	Endothelial cell count (cells/mm^2^) - presentation	BCVA logMar - first month	Endothelial cell count (cells/mm^2^) - first month	BCVA logMar - sixth month	Endothelial cell count (cells/mm^2^) - sixth month
F	ABMD	0.6	3,000	0.0	2,990	0.0	2,990
F	ABMD	0.3	2,940	0.0	2,970	-0.1	2,980
F	ABMD	0.2	2,920	-0.1	2,910	-0.1	2,980
F	ABMD	0.0	2,700	0.0	2,700	0.0	2,650
M	ABMD	0.2	2,450	-0.1	2,500	0.0	2,400
F	ABMD	0.5	2,450	0.2	2,350	0.2	2,400
F	Traumatic	0.3	2,450	0.0	2,350	0.0	2,400
F	Traumatic	0.2	2,470	-0.1	2,500	0.0	2,500
M	Traumatic	0.3	2,680	0.0	2,700	0.0	2,600
M	Traumatic	0.5	2,500	0.3	2,450	0.2	2,500
M	Traumatic	0.2	2,650	0.0	2,680	0.0	2,680
F	ABMD	0.2	2,550	0.2	2,500	0.0	2,650
F	ABMD	0.3	2,770	0.0	2,750	0.0	2,770
F	Traumatic	0.8	2,850	0.0	2,850	0.0	2,830
M	ABMD	0.6	2,800	0.2	2,750	0.0	2,800
F	Traumatic	0.5	2,550	0.2	2,550	0.0	2,600
F	Traumatic	0.3	2,900	0.2	2,860	0.0	2,880
F	ABMD	0.2	2,850	0.2	2,860	0.2	2,850
F	ABMD	0.2	2,760	0.0	2,700	-0.1	2,680
M	ABMD	0.2	2,880	0.0	2,850	0.0	2,860
M	Traumatic	0.2	3,000	0.0	3,000	0.0	3,000

**Figure 1:**
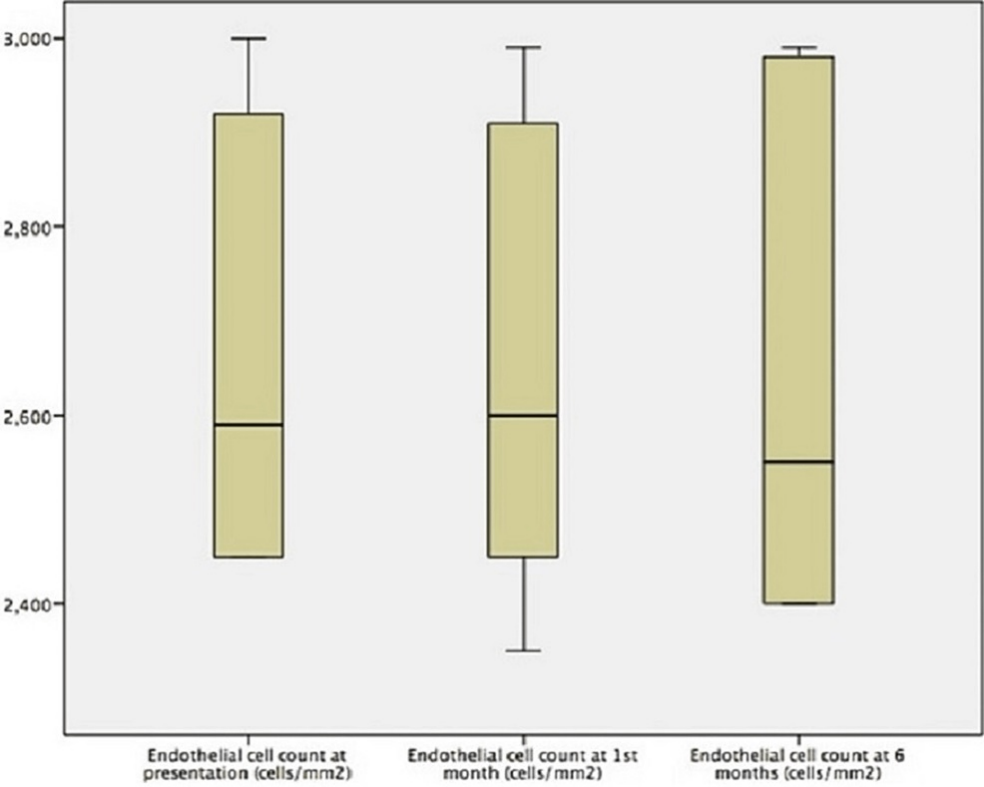
Median and range of endothelial cell count at the three time points of examination.

At the one-month follow-up, all 21 patients had a full recovery from the initial episode with complete re-epithelization of the defected area and resolution of symptoms. The mean BCVA improved to 0.06 logMAR units (SD = 0.12), and the mean endothelial cell count was 2,703 cells/mm^2^ (SD = 205). At the six-month follow-up, there was only one recurrence of corneal erosion in a patient with post-traumatic RCES. The recurrence occurred when the subject (a 68-year-old male) sustained further trauma due to intense eye rubbing at four months while on no treatment. No drop in endothelial cell count was observed. He was managed conservatively with topical lubricants and antibiotics by the Accident and Emergency Department where he presented after the recurrence. All other patients remained symptom-free with an intact corneal epithelium and were classified as successful treatment. The mean BCVA and endothelial cell count at six months were 0.01 logMAR units (SD = 0.08) and 2,714 cells/mm^2^ (SD = 201), respectively. The mean BCVA at presentation was significantly statistically different compared to the other two time points of examination (p < 0.0001, t-test) one and six months after initiation of treatment, demonstrating the acute impairment of vision and subsequent significant improvement with the proposed treatment. However, no difference in BCVA was observed between one and six months post-treatment. The median values and range of endothelial cell count at presentation, first-month, and sixth-month follow-up are depicted in Figure 1. Of note, the endothelial cell count remained largely stable in all subjects from the acute event to the completion of follow-up, with the means not being statistically significantly different at p < 0.05, indicating that neither the acute event of the epithelial disruption nor the treatment regimen applied had any detrimental effect on the corneal endothelium. No complications in terms of corneal scarring or infective keratitis were observed.

Furthermore, within-subject ANOVA showed no effect of sex, age, and diagnosis on BCVA and endothelial cell counts, an observation that was maintained throughout all other visits.

## Discussion

RCES is a common reason for attendance to eye casualty and may cause much frustration to the patient and the treating ophthalmologist because of its recurrent and variable clinical course. Patients with traumatic recurrent erosion syndrome are expected to be less symptomatic in the long term and less likely to suffer recurrences than those with ABMD [10]. Many treatment strategies which aim at regenerating or repairing the adhesion between the epithelium and the anterior stroma are currently available and range from conservative to surgical [9,11]. They should be tailored to each patient depending on number of recurrences, individual level of discomfort and risk of complications, which include corneal haze and scarring with subsequent decreased vision, and infective keratitis [5]. All different treatment modalities for RCES, either conservative or surgical, report various degrees of success; however, no definitive management algorithm has been agreed upon, and there is lack of randomized controlled trials comparing the existing management measures [11].

NaCl 5% ophthalmic ointment is a hypertonic agent which is used to normalize the cornea when the abnormal or irregular epithelium is edematous. Its mode of action in RCES is thought to be the exertion of an osmotic action through the tear film, thus reducing the associated corneal edema and subsequently promoting adhesion between the epithelium and the basement membrane. It has been previously used as a conservative prophylactic treatment for non-acute RCES with good results [8].

All patients experienced resolution of symptoms with complete re-epithelization after one month of treatment with NaCl 5% ointment. The lack of further complaints remained at six months in all but one patient. The patient experienced a recurrence of his symptoms after sustaining renewed trauma associated with excessive eye rubbing. It should be noted that NaCl 5% ointment does not provide causal treatment repair to the associated basement membrane dystrophy and does not lead to the disappearance of microcysts and macrocysts. However, it provides lubrication and improves the adhesion of newly formed epithelium due to the reduction of corneal edema associated with the epithelial defect of recurrent erosion.

Previously, it has been postulated that endothelial cell count can be influenced by the use of NaCl 5% [12,13]. This notion has been confirmed by the results of our study as well as with the density of endothelial cells remaining unaffected following treatment.

Our study shows that NaCl 5% ointment is a safe alternative for the treatment of RCES of both traumatic and idiopathic etiology. As previous studies have established that lubricating ointment in addition to standard treatment following traumatic corneal erosions led to increased symptoms of recurrent corneal erosion [14], we aimed to provide data on the use of NaCl 5% ointment as the sole treatment for the acute and recurrent forms of corneal erosion.

Limitations of our study are the small number of patients and the relatively short follow-up. A prospective study comparing patients treated with hypertonic saline ointment with those managed with other available modalities, including a control group, is warranted to better assess the safety and efficacy of this treatment.

## Conclusions

NaCl 5% ophthalmic ointment applied twice daily for 30 days following the acute event appears to be a safe and effective treatment option for RCES both from traumatic and ABMD etiology. Future studies can examine the epithelial healing time and the impact of our treatment protocol on services, such as the number of eye casualty attendances for RCES and the impact on patient satisfaction and lifestyle. Overall, further trials are needed to establish the benefits of standardized treatment regimens for recurrent corneal erosions.

## References

[1] Hansen E (1872). On den intermitterende keratitis visicularis neuralgica af traumatisk oprindelse. Hospitalis-Tidende.

[2] Wood TO (1984). Recurrent erosion. Trans Am Ophthalmol Soc.

[3] Dursun D, Kim MC, Solomon A, Pflugfelder SC (2001). Treatment of recalcitrant recurrent corneal erosions with inhibitors of matrix metalloproteinase-9, doxycycline and corticosteroids. Am J Ophthalmol.

[4] Bernauer W, De Cock R, Dart JK (1996). Phototherapeutic keratectomy in recurrent corneal erosions refractory to other forms of treatment. Eye (Lond).

[5] Watson SL, Barker NH (2007). Interventions for recurrent corneal erosions. Cochrane Database Syst Rev.

[6] Brown N, Bron A (1976). Recurrent erosion of the cornea. Br J Ophthalmol.

[7] Kenyon KR (1979). Recurrent corneal erosion: pathogenesis and therapy. Int Ophthalmol Clin.

[8] Hykin PG, Foss AE, Pavesio C, Dart JK (1994). The natural history and management of recurrent corneal erosion: a prospective randomised trial. Eye (Lond).

[9] Miller DD, Hasan SA, Simmons NL, Stewart MW (2019). Recurrent corneal erosion: a comprehensive review. Clin Ophthalmol.

[10] Heyworth P, Morlet N, Rayner S, Hykin P, Dart J (1998). Natural history of recurrent erosion syndrome--a 4 year review of 117 patients. Br J Ophthalmol.

[11] Ewald M, Hammersmith KM (2009). Review of diagnosis and management of recurrent erosion syndrome. Curr Opin Ophthalmol.

[12] Bachmann BO, Roessler K, Tourtas T, Weller J, Schlotzer-Schrehardt U, Kruse F (2014). The influence of hypertonic sodium chloride eye drops on the early postoperative course after Descemet membrane endothelial keratoplasty (DMEK). Invest Ophthalmol Vis Sci.

[13] Tsatsos M, Savant V, Prydal J (2011). Efficacy and side-effects of hypertonic sodium chloride ointment (5%) in the treatment of recurrent corneal erosions. Invest Ophthalmol Vis Sci.

[14] Watson SL, Lee MH, Barker NH (2012). Interventions for recurrent corneal erosions. Cochrane Database Syst Rev.

